# Vocal Tract Images Reveal Neural Representations of Sensorimotor Transformation During Speech Imitation

**DOI:** 10.1093/cercor/bhx056

**Published:** 2017-03-18

**Authors:** Daniel Carey, Marc E. Miquel, Bronwen G. Evans, Patti Adank, Carolyn McGettigan

**Affiliations:** 1 Department of Psychology, Royal Holloway, University of London, London TW20 0EX, UK; 2 Combined Universities Brain Imaging Centre, Royal Holloway, University of London, London TW20 0EX, UK; 3 The Irish Longitudinal Study on Ageing (TILDA), Department of Medical Gerontology, Trinity College Dublin, Dublin, Ireland; 4 William Harvey Research Institute, Queen Mary, University of London, London EC1M 6BQ, UK; 5 Clinical Physics, Barts Health NHS Trust, London EC1A 7BE, UK; 6 Department of Speech, Hearing & Phonetic Sciences, University College London, London WC1E 6BT, UK; 7 Institute of Cognitive Neuroscience, University College London, London WC1N 3AR, UK

**Keywords:** FMRI, learning., rtMRI, sensorimotor transformation, speech

## Abstract

Imitating speech necessitates the transformation from sensory targets to vocal tract motor output, yet little is known about the representational basis of this process in the human brain. Here, we address this question by using real-time MR imaging (rtMRI) of the vocal tract and functional MRI (fMRI) of the brain in a speech imitation paradigm. Participants trained on imitating a native vowel and a similar nonnative vowel that required lip rounding. Later, participants imitated these vowels and an untrained vowel pair during separate fMRI and rtMRI runs. Univariate fMRI analyses revealed that regions including left inferior frontal gyrus were more active during sensorimotor transformation (ST) and production of nonnative vowels, compared with native vowels; further, ST for nonnative vowels activated somatomotor cortex bilaterally, compared with ST of native vowels. Using test representational similarity analysis (RSA) models constructed from participants’ vocal tract images and from stimulus formant distances, we found that RSA searchlight analyses of fMRI data showed either type of model could be represented in somatomotor, temporal, cerebellar, and hippocampal neural activation patterns during ST. We thus provide the first evidence of widespread and robust cortical and subcortical neural representation of vocal tract and/or formant parameters, during prearticulatory ST.

## Introduction

Speech imitation is a complex and multistage process that requires the interaction of both sensory and motor systems, such that acoustic inputs can be processed, transformed to target motor outputs, and articulated as speech (see Guenther 2006; Bohland et al. 2010; Guenther and Vladusich 2012). Early accounts proposed that the perceptual components of this multistage process hinge upon central speech representations that occur at the subphonemic level; these representations would code for the motor effectors necessary for speech articulation, during initial perception of the speech signal (Liberman et al. 1967). The predictions of this motor theory of speech perception have received mixed support (e.g., Pulvermüller et al. 2006; D'Ausilio et al. 2009; Möttönen and Watkins 2009; cf., Scott et al. 2000). Nevertheless, more recent models of speech have further sought to link perception and production, by charting the contributions of sensory and motor representations to both perceptual and articulatory processes (e.g., Du et al. 2014; Correia et al. 2015; Evans and Davis 2015). Current understanding points toward a multistage unfolding of speech representations, with sensorimotor transformation (ST) identified as a critical prearticulatory component (Cogan et al. 2014; Leonard et al. 2016).

ST reflects the process of converting from an input speech acoustic signal (heard or imagined) to the phonemic and motor representations needed to execute the articulatory gestures that enable production of the perceived speech input (Wilson and Iacoboni 2006; Mesgarani et al. 2014; Parker-Jones et al. 2014). Current data suggest that neural representations during ST reflect a unified coding of both the phonemic identity of an utterance and the specific motor outputs required to repeat it (Cogan et al. 2014). However, much controversy has surrounded claims about the neural substrates that support ST processes. While some have argued for a central role of posterior Sylvian regions (Sylvian-parietal-temporal) in transforming from sensory representations to motor output (Hickok and Buchsbaum 2003; Hickok and Poeppel 2007; Hickok et al. 2009; Hickok 2012), others have failed to support these claims (Parker-Jones et al. 2014) or have suggested the involvement of more widespread sensory and motor regions (Cogan et al. 2014; Simmonds et al. 2014b).

Given the multimodal nature of speech, a key challenge for research is to map insights from neural data onto speech articulatory behavior (Bouchard et al. 2016), and onto parameters of speech acoustics (Mesgarani et al. 2014; Cheung et al. 2016). Developing a mechanistic understanding of speech thus requires that we link the acoustics of speech input and the subsequent actions of the speech motor effectors (i.e., lips, tongue, larynx) to the central brain representations that govern ST and speech production. With this approach, we can more comprehensively explain speech as an audiomotor behavior, with respect to the functional brain representations that support that behavior (see Carey and McGettigan 2016). Yet to date, few studies have sought to explore acoustic and articulatory facets of speech representation for ST, or articulatory aspects of speech representation during production itself (though see Bouchard et al. 2016).

While the acoustics of speech have been measured and quantified for decades, a fundamental difficulty has been probing the articulatory basis of speech production directly. Real-time MR imaging (rtMRI) of the vocal tract during speech and related tissue analysis techniques now afford a noninvasive way to measure articulatory markers of speech production directly from the vocal tract (Scott et al. 2014; Lingala et al. 2016; Toutios and Narayanan 2016). Such data and methods are amenable not only to making direct measurements of articulatory performance (e.g., tracking in-frame articulator position), but also to offline integration with other MR image-based methods, such as functional MRI (fMRI). More specifically, in seeking to unite neural and articulatory data, as well as acoustic properties of speech, multivariate techniques such as representational similarity analysis (RSA) (Kriegeskorte et al. 2008; Kriegeskorte and Kievit 2013) provide a highly insightful means by which to integrate these cross-modal data sources and assay neural representations for speech. Critically, RSA enables the comparison of distinctly different sources of data (e.g., fMRI, vocal tract images, speech acoustics) in representational terms, based on the expression of the data within an amodal dissimilarity space (Kriegeskorte et al. 2008). The representational basis of articulatory and/or speech acoustic information at the neural level can then be probed by comparing and quantifying the degree of relatedness between these dissimilarity patterns. RSA can thus support data-driven approaches to modeling neural representations of vocal tract behavior and/or speech acoustics that may emerge during ST and speech imitation. Moreover, the potential to use searchlight analyses, where representations are tested iteratively at adjacent locations within a brain volume (Kriegeskorte et al. 2006), offers a powerful method for probing the neural substrates that support these speech representations (e.g., Evans and Davis 2015).

In the current study, we explored the representational bases of speech ST and production, by combining neural (fMRI), articulatory (rtMRI of the vocal tract) and acoustic (speech spectra and formants) data, to offer a multidimensional account of speech. Monolingual adult participants trained on imitating an unrounded native vowel and a similar nonnative vowel that required lip rounding. Later, participants provided fMRI data as they listened to and then subsequently imitated these trained vowels, as well as a further untrained pair. Importantly, an event-related rapid-sparse fMRI task design enabled us to probe both ST and imitation, by sampling the blood oxygen level-dependent (BOLD) response during both task phases, per trial. In separate runs interleaved with fMRI blocks, rtMRI allowed us to capture lip dynamics for rounded versus unrounded vowels directly from participants’ vocal tracts (see Methods). We first tested whether nonnative lip dynamics were acquired after training, and whether trained dynamics extended to the untrained nonnative vowel. We then tested condition-wise representational patterns in our fMRI data against models built from vocal tract MR images during speech. This allowed us to probe neural data from speech imitation and ST with data-driven test models that reflected the position of the articulators as participants spoke each vowel. We further constructed 2 test models that described the relatedness between the vowel stimuli, based on the spectral properties of the stimulus acoustics, and based on the stimulus distances in formant space, respectively. We predicted that, over the 4 vowel conditions, models of articulator position derived from vocal tract images would correlate with condition-wise searchlight fMRI data representational patterns: (1) within a brain mask reflecting regions where there was univariate activation for ST over all vowels (i.e., all ST > rest) and (2) within a brain mask reflecting regions where there was univariate activation for imitation over all vowels (i.e., all imitation > rest). We further expected that models of speech stimulus spectra and formant distances would correlate with neural patterns for ST across conditions, within searchlights of regions active during ST for all sounds (i.e., all ST > rest).

## Materials and Methods

### Participants

Participants were 24 healthy right-handed monolingual female volunteers (mean age ± standard deviation [SD]: 25.9 ± 5.9; range: 19–38), free from any history of language or hearing difficulties. All were native British English speakers; none had proficiency in any nonnative language beyond UK GCSE or equivalent. Given that a female talker provided our stimuli (see below), we tested female participants only, in order to avoid potential gender confounds in imitation accuracy. All provided written informed consent in line with local ethics and MRI protocols. The study was approved by the Ethics Committee at the Department of Psychology, Royal Holloway, University of London.

### Stimuli

**Figure 1.**
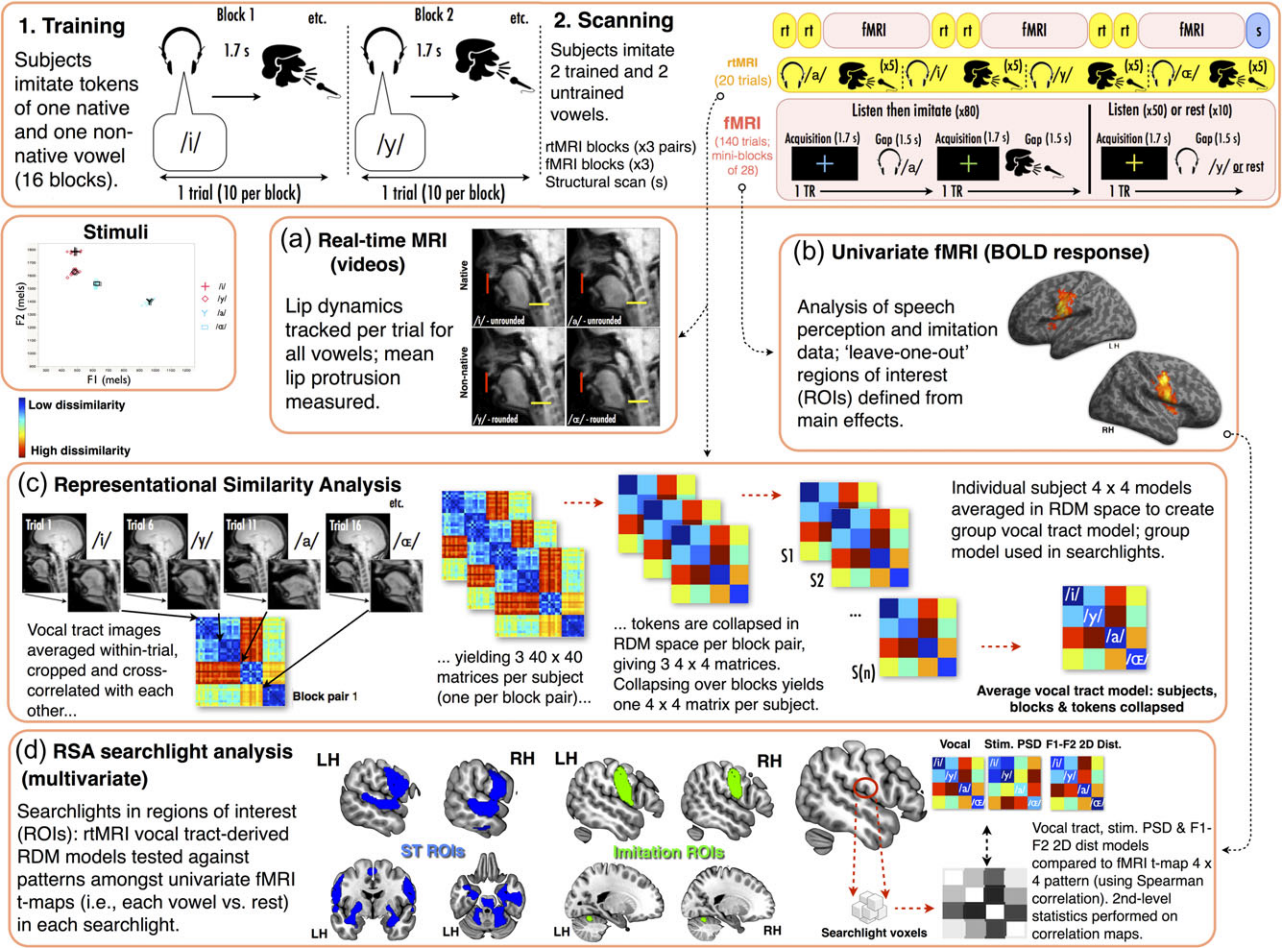
Overview of experimental paradigm and analysis framework. *Upper row.* (1) Participants trained on imitating one native and one nonnative vowel in blocks; all 10 tokens from a single category (e.g., /i/ or /y/) were imitated in randomized order in a given block (stimuli F1 and F2 are plotted in mel space—see lower inset). (2) Training was followed by scanning, during which participants imitated the trained pair and a further untrained pair. Scans comprised 3 fMRI blocks (140 trials, ~12 min), each preceded by a pair of rtMRI blocks (40 trials, ~3 min). *Data analyses* (*a*–*d*)*.* (*a*) rtMRI data were analyzed with the Matlab toolbox of Kim et al. (2014), yielding measures of lip position per vowel (red trace on panels). (*b*) fMRI data were first analyzed with SPM, with contrasts specified for main effects (Imitation > rest; Listen preimitate > rest) (surface shown presents all imitation > rest second-level contrast, for illustrative purposes). Further contrasts were specified for each vowel > rest, for listen preimitation and imitation stages of the task. ROIs were defined with a Jackknifed “leave-one-out” procedure using the listen preimitate or imitation main effects (all vowels > rest). (*c*) rtMRI images frames were first averaged within a single trial (using the method of Scott et al. 2013). Images were then masked with the RSA toolbox, restricting FOV to the vocal tract. Masked images were cross-correlated on a trial-wise basis, creating three 40 × 40 RDMs (one per rtMRI block pair). Converting RDMs from correlation distance to *z*-score (with Fisher transform), each RDM was reduced to 4 × 4 matrix, and 4 × 4 matrices were averaged to give a single 4 × 4 matrix per subject. Single-subject 4 × 4 models were averaged to produce a full cohort 4 × 4 average model. Single-subject and full cohort models were used in searchlight analyses. (*d*) Schematic of the RSA searchlight procedure. Jackknifed ROIs constrained the searchlight analyses to regions active for imitation (all imitation > rest) and ST (all listen preimitate > rest). In each searchlight, the RDM pattern from the t-maps for the vowel conditions was correlated with the vocal tract image-derived model, the stimulus PSD acoustic-derived model, or the stimulus F1–F2 2D Euclidean distance derived model (see Materials and Methods).

Stimuli were steady-state front vowels (mean duration [ms] ± SD: 782 ± 72), produced by a female British English phonetician. Vowels belonged to 4 categories: 2 native to English (/i/, /a/, both unrounded) and 2 nonnative (/y/, /ɶ/, both rounded). Here, the native/nonnative distinction maps onto the articulatory feature of lip rounding, as rounding of front vowels is nonnative to English (see Wells 1970). Ten tokens were included per category (40 stimuli in total; see Fig. 1).

For each category, initial raw recordings comprised 20 exemplars; we converted the F1 and F2 formant measurements to mels (O'Shaughnessy 1987), and selected the 10 tokens per category as follows: first, we calculated the median of F1 and F2 values across 10 potential tokens (formants were measured over the full vowel duration using Praat software; Boersma and Weenink 2016). Second, we calculated the 2D Euclidean distance between the category median (F1 and F2) and each token (F1 and F2). Third, we calculated the SD of the 2D Euclidean distances for that category. Finally, we matched each of the categories as closely as possible for the SD of token distances to their respective category median (replacing tokens with other exemplars in some instances). Stimuli were selected in this systematic fashion to ensure that variability of tokens within each category was controlled as carefully as possible across each of the 4 vowels. Stimuli were scaled to equal total RMS amplitude in Adobe Audition CS 5.5 (Adobe Systems Inc.).

For use in the scanner, stimuli were parametrically equalized (in Adobe Audition; filter CF: 3.5 kHz; 10 dB gain; Q factor = 2), filtered with earbud-specific parameters for use with Sensimetrics earbuds (S14; Sensimetrics Corp.), and amplified by +6 dB with Adobe Audition. Parametric equalization and amplification were applied to ensure that all vowels were clearly distinguishable against continuous rtMRI acquisition noise.

In addition to the natural vowel tokens, we created spectrally rotated versions of each of the 40 stimuli. These served as an acoustic baseline in the fMRI task (10 trials per run) that preserved the spectro-temporal complexity of speech; responses to this condition are not reported here.

### Behavioral Training Procedure

Participants completed a language background questionnaire including proficiency estimates for any languages they had learned. All testing took place in a sound attenuated booth. All experiments were presented in Matlab (2014a, the Mathwork) using the Psychophysics toolbox (Kleiner et al. 2007). Audio stimuli were presented through Sennheiser HD 201 headphones (Sennheiser electronic GmbH & Co. KG).

Participants were randomly assigned to 1 of 2 counterbalanced training conditions: the first group received training on the vowel pair /i/–/y/ and the other group trained on the /a/–/ɶ/ pair. Participants watched a 2-min training video, featuring the same phonetician as heard in the stimuli. The video included: repetitions of the rounded and unrounded vowels; instructions on nonnative lip rounding; multiple camera angles, with close-up front and profile views of the rounded and unrounded dynamics (with and without phonation). Two versions of the video were produced, one for each training pair (i.e., /i/–/y/ or /a/–/ɶ/), that differed only in the frames where vowels/lip rounding were demonstrated. The training video helped to assure that participants were presented with clear and accessible audio-visual instructions as to how the lip protrusion should be performed. Moreover, the use of the videos ensured that the instructions were always consistent across subjects and between the 2 training conditions (with the exception of the frames where specific vowels were demonstrated).

Participants then completed the training (16 blocks of 10 trials; 8 blocks per vowel). In a given block, the task was to imitate all 10 tokens from a single category as accurately as possible. Each trial began with a visual prompt (“Listen”) at the upper left of the screen, and delivery of one token from the category for that block. At stimulus offset, the upper left visual prompt was replaced (“Pause...”) for 1.7 s, followed by a 2 s repeat window (“Repeat”), during which participants imitated the vowel. The next trial began after 2 s had elapsed. Block order for vowel category was pseudorandomized, with the constraint that the same vowel category repeated no more than once on adjacent blocks. Imitations were recorded with a condenser microphone (Røde NT1-A), digitized in Matlab, and saved as separate .wav files per trial. At the beginning and at the end of the session, participants made “same or different” 2-alternative forced choice perceptual judgements on pairs of exemplars from within and across the 4 stimulus categories. Mean *d*′ scores showed high accuracy (i.e., *d*′ > 2) in discrimination for all pairs, before and after training.

### MRI Procedure

Data were acquired on a 3 T Siemens Tim Trio with a 12-element headcoil (fMRI and rtMRI) and 3-element neck array (rtMRI) (Siemens). All stimuli were delivered through MR-compatible earbuds; speech was recorded per run with a fiber-optic microphone (FOMRI-III; OptoAcoustics Ltd). All stimuli were presented via the Psychophysics toolbox running in Matlab, with back projection for presentation of visual stimuli.

After completing the imitation training on one pair of vowels (see above), we presented participants with both pairs of vowels during fMRI and rtMRI, giving a 2 (training) × 2 (native/nonnative) design. This enabled us to probe training outcomes for both nonnative vowels using rtMRI, and to test the representational basis of trained and untrained vowel conditions in fMRI data using univariate analyses and RSA. A pair of rtMRI runs (65 s each) was presented before each of the 3 fMRI runs (~12 min each; total scanning time ~60 min; Fig. 1).

fMRI acquisition entailed a rapid-sparse, event-related protocol, where auditory stimuli and speech production events were timed to occur during short silent periods between acquisition of whole-brain volumes. Each listen-imitation trial occurred over 2 acquisition + silent gap periods; participants listened to a particular vowel, and imitated it when cued after the next acquisition. This enabled us to capture BOLD activation reflecting ST and the subsequent vowel imitation. Listen only and rest trials occurred in a single acquisition + silent gap period (see Fig. 1). In the following, we distinguish listening that entailed ST from passive listening, as “listen preimitate” and “listen only”, respectively. Five event types were thus presented during fMRI: listen preimitate (vowels), imitation (vowels), listen only (vowels), listen only (spectrally rotated vowels), and rest.

fMRI trials for listen preimitate and imitation were cued as follows. At the onset of the first acquisition, a blue fixation cross cued that the trial would require vowel imitation. Vowel stimuli were presented in the silent period after this first acquisition (i.e., onset of ST); stimulus onsets were jittered variably (50–500 ms) from the start of the silent gap. At the offset of the next acquisition, the blue fixation cross changed to green, cueing the participant to imitate the vowel.

Listen only (speech and audio baseline) trials and rest trials were cued at acquisition onset with a yellow fixation cross that remained for the trial duration (one acquisition + silent gap period); stimuli were delivered with onsets jittered variably as above. Participants were instructed to remain alert during listen trials and not to produce any speech. Five miniblocks of 28 trials (16 listen then imitate, 8 listen only, and 2 each of rest and auditory baseline) were presented per fMRI run (140 trials total: 80 listen and production, 40 listen only, and 10 each of rest and auditory baseline). Trial order was randomized separately for each miniblock.

fMRI data were 3D echo-planar images (EPIs) collected with rapid-sparse acquisition, voxel size 3 mm isotropic, flip angle 78°, slice gap 25%, echo time (TE) 30 ms, vol. acquisition time 1.7 s, and inter-scan silent period 1.5 s. A 3D T_1_-weighted MP-RAGE scan was acquired for EPI image alignment and spatial normalization, voxel size 1 mm isotropic, flip angle 11°, TE 3.03 ms, time repetition (TR) 1830 ms, and image matrix—256 × 256.

rtMRI blocks comprised pairs of 65 s runs. Within each run, participants imitated all 4 vowel categories, with each vowel category delivered in a miniblock of 5 consecutive trials. The order of vowel miniblocks was pseudorandomized separately per run. Five different tokens per category were presented in a run, so that over a pair of runs, participants imitated all 10 tokens from each given category. Each trial began with delivery of a vowel stimulus and a visual prompt (“Listen”), followed by a prompt to imitate (“Repeat”).

Real-time data were fast gradient echo images; flip angle: 5°; TE/TR: 1.25/125 ms; GRAPPA factor 2; partial-Fourier: 75%; FOV: 220 × 274 mm^2^; 2.5 × 2.5 × 10.0 mm^3^ spatial and 125 ms temporal resolution (8 frames per second [f.p.s.]). Pilot experiments showed that we could obtain adequate numbers of frames during steady-state phonation when sampling at 8 f.p.s., to enable us to index articulator positioning for the vowels. Further, our images achieved good whole-vocal tract spatial resolution, and were hence suited to use in processed form within RSA models (see below).

### Data Processing and Analyses

#### Functional MRI

fMRI data were preprocessed and analyzed in SPM8 (Wellcome Trust Centre for Neuroimaging, UK). Functional images for each run were realigned, and the mean functional image co-registered with the anatomical scan. For each run, we set a motion criterion such that all acquisitions had maximum translations that were less than a single dimension of one voxel (i.e., for any single acquisition, the total translation over the 3 axes was <3.0 mm, relative to the mean functional image). Only one participant exceeded this criterion, and was excluded from further analyses. In practice, we found that translations about the *z*-axis were most common, and of 1–2 mm magnitude. After image realignment and co-registration, location of the anterior commissure (AC) was determined manually from the anatomical scan. Structural and functional images were then reoriented so the origin of each image matched the AC, prior to spatial normalization. Functional images were spatially normalized with parameters derived from the unified segmentation of the anatomical image, with resampling to 2 mm isotropic voxel dimensions. Smoothing was applied with an 8 mm FWHM Gaussian kernel.

At first-level analysis, each condition was modeled with a separate regressor of event onsets in a general linear model, convolved with a canonical hemodynamic response function (HRF); rest was modeled implicitly. Event onsets for listen only trials and listen preimitate trials were modeled using the onset time of the audio stimulus. Event onsets for speech imitation were modeled using the onset of the cue to imitate (i.e., crosshair color change at acquisition offset). The 6 motion parameters (translations and rotations about the *x*, *y*, and *z* axes), the run mean image, and onsets of any events that reflected in-scanner task errors were included as per-run regressors of no interest. We assured noncollinearity of regressors in the analyses via our task design: imitation trials could be followed by listen only or rest trials, or a further imitation trial, so participants could never accurately predict the next trial type. Additionally, we jittered the preimitation stimulus onsets variably across trials (50–500 ms postacquisition offset; see above).

We excluded one fMRI dataset due to motion artifact, and analyzed fMRI data from 23 participants. Error trials (e.g., no speech on an imitation trial) were flagged by comparing in-scanner audio recordings to saved stimulus logs (group mean task accuracy: >96% per block), with the scan events reflecting those trials flagged and included as regressors of no interest per run (see above).

First-level t-contrasts of interest modeling effects of each of the 4 vowels (vs. rest) were specified for listen preimitation and imitation; the t-maps for listen preimitation and imitation were entered into 2 × 2 univariate analyses (factors: native/nonnative; trained/untrained) and RSA analyses (see below). First-level t-contrasts (vs. rest) were also specified for each main effect of listen preimitate, imitation, and listen only. To constrain analyses to regions critical to speech perception and production, we a priori elected to confine RSA searchlights to regions of interest (ROIs) comprising areas that were active in listen preimitation (vs. rest), and imitation (vs. rest; see RSA Analyses).

#### Real-Time MR Imaging

Real-time image dynamics were analyzed using a custom Matlab toolbox (see Kim et al. 2014). Output lip and larynx co-ordinates for each trial were saved for offline averaging and analyses. Within each block pair, we averaged the *x* co-ordinates at the steady-state frames per vowel. Lip *x* co-ordinate difference scores (unrounded – rounded) were calculated for each vowel pair, for analysis at group level. These measures expressed the relative difference in the *x* co-ordinate when comparing each native vowel with its nonnative counterpart. Difference measures were initially calculated within block pair and then later averaged, so as to minimize the possibility that head movement between blocks biased the *x* co-ordinate. The use of a difference score in particular also helped to account for slight movements due to head motion, which tend to be consistent across the vowels in a single block pair. During rtMRI, we sampled the frames corresponding with the steady-state portion of the articulation. We appreciate that “dynamics” might imply that the dependent variable incorporates a temporal dimension reflecting movement during the measured frames; here, we intend it with respect to a process that is overall dynamic (given that the lips had to move from a stable resting location to the appropriate position during articulation), but based on a dependent measure that samples the position of the articulators in time, once a stable arrangement has been reached. We should note that the measure does include a temporal dimension—several frames were averaged over within each trial to produce the estimate of lip position—albeit the position of the articulators was largely stable during those frames.

Per trial, we averaged the consecutive real-time frames in the middle of the trial where articulator position was stable (minimum 2 frames per trial; typically 4–6 frames), using the adaptive averaging procedure of Scott et al. (2013). A pixel intensity-based rigid body translation was then applied (using in-house Matlab routines), aligning images from trials of the same category that were collected across separate runs of a block pair. These averaged and aligned images were used in the construction of vocal tract derived test models for RSA analyses (see below).

### Representational Similarity Analysis

RSA provides an analysis framework with which to evaluate the neural representation of specific conditions. This can be achieved by comparing patterns of relationships for neural activation across conditions with predefined test models that reflect a predicted pattern of condition-wise relationships (Kriegeskorte et al. 2008; Kriegeskorte and Kievit 2013). The extent to which the pattern of neural activation relates to the test model pattern may then be evaluated statistically, to quantify the representational basis of the expected model at the neural level.

Relationships amongst conditions in RSA are expressed as the correlation distance (i.e., 1 – Pearson Product moment correlation) between all possible condition pairs for a given data type (e.g., fMRI activation maps). This yields a Representational Dissimilarity Matrix (RDM), where each RDM cell reflects the extent of dissimilarity between a pair of conditions (0 → +2, where 0 reflects null dissimilarity, i.e., perfect correlation). An RDM derived from neural data can be compared with a given test RDM with a Spearman correlation; this provides a test of the extent to which the neural pattern of relationships correlates with the expected model pattern.

Here, we performed RSA searchlight analyses of neural activation within ROIs (Kriegeskorte et al. 2006). We compared the condition-wise patterns amongst fMRI t-maps for vowel ST (i.e., listen preimitate trials) and for vowel imitation, to the test RDM patterns that we derived from images of the vocal tract during speech. In this way, we could test the prediction that the neural representation of vowel ST and production would reflect the pattern of dissimilarity amongst conditions that emerged based on the physical positions of participants’ vocal tracts during imitation of vowel categories.

Additionally, we defined 2 further test RDMs built from (1) the spectral properties of the vowel stimulus acoustics and (2) the distances between the stimuli in vowel formant space. This enabled us to probe whether representations at the neural level during speech ST would reflect dissimilarity patterns that related to the raw acoustic input that the ST was initially based on, or to a more abstracted perceptual representation of the vowels.

#### Vocal Tract RDM Construction

Using the within-trial adaptively averaged rtMRI images from each subject, we created subject-wise RDMs using the RSA toolbox (www.mrc-cbu.cam.ac.uk/methods-and-resources/toolboxes/). Per subject, these RDMs comprised correlation distances (1 – correlation coefficient) between the vocal tract images from the trials for each vowel condition (i.e., the imitations produced during rtMRI runs).

To create the vocal tract RDMs, the averaged rtMRI images were first vectorised in Matlab; bespoke masking was then applied to each subject's data, reducing the field-of-view for each trial-wise average rtMRI image (to exclude non vocal tract tissue). For a given subject and real-time block pair, we cross-correlated every masked trial-wise image with all other images; this yielded one 40 × 40 matrix per block pair (3 of these matrices in total per subject; Fig. 1*c*, second left). Within each 40 × 40 matrix per subject, we converted every correlation distance to a correlation coefficient (i.e., by subtracting each correlation distance from 1). Next, we Fisher *z*-transformed each of the correlation coefficients, which gave three 40 × 40 matrices comprising *z*-transformed values. We then averaged together the *z*-transformed values that reflected the trial by trial comparison of the items within a given vowel category, and also between each pair of vowel categories (matrix 0 diagonals were excluded from averaging to avoid bias). This yielded 3 summary 4 × 4 matrices per subject (Fig. 1*c*, middle); we then averaged these 3 matrices within-subject (i.e., collapsing blocks), and transformed back to correlation distance (i.e., reversing the *z*-transform procedure above, and subtracting the correlation coefficients from 1) to give an overall vocal tract RDM for each participant. Finally, we averaged the *z*-transformed subject-wise 4 × 4 matrices across the full cohort, and transformed back to correlation distance: this produced a single grand average 4 × 4 RDM that described the overall pattern of dissimilarity between the 4 vowels that subjects produced, based on the full cohorts’ real-time vocal tract data (see Fig. 1*c*).

We used the group average vocal tract model as input to searchlight analyses of each subject's fMRI t-maps across conditions (vs. rest). Additionally, we used each subject's own 4 × 4 vocal tract image-derived RDM as a bespoke model, to conduct searchlights of the fMRI t-maps across conditions.

#### Stimulus Acoustic RDM Construction

We processed each audio stimulus presented to participants, by extracting 120 ms of audio centered on the midpoint of each stimulus sound file. For each of the sound files, we derived the power spectral density (PSD) matrix of the excised segment (using a Goertzel discrete Fourier transform spectrogram algorithm in Matlab; range 0.1–5000 Hz; 260 number of Fourier transform sampling points; 0.1 Hz increment) (see Carey and McGettigan 2016). Each PSD matrix was used as input to the RSA toolbox and cross-correlated over all possible pairs (yielding a 40 × 40 matrix); we averaged RDM matrix values within and across category tokens with a similar procedure as for rtMRI images (above) to create a summary 4 × 4 RDM. Rank correlations comparing the stimulus derived RDM to the vocal tract image-derived RDM showed that the models were not correlated (Spearman *ρ* and Kendall *τ* both <0.1, *P* > 0.9).

#### Vowel 2D Euclidean Distance RDM Construction

In addition to the stimulus acoustic model based on the PSD of the vowel stimuli, we constructed a model based on the 2D Euclidean distances between the first and second formants (F1 and F2) of the stimuli, in Mel space. As outlined in Stimuli (see above), the first and second formants defined the stimulus categories within vowel acoustic space; moreover, F1 and F2 serve as the acoustic correlates of tongue height and frontness, respectively. Thus, we wished to test whether a representational model that reflected the acoustic distances amongst the stimulus categories—and that was more broadly indicative of acoustic correlates of articulator position—was represented at a neural level. To construct the model, we calculated the 2D Euclidean distance (in Mels) between the F1 and F2 of each possible pair of stimuli. This afforded a 40 × 40 matrix of 2D Euclidean distance values; we rank transformed each distance value to lie between 0 and 1 (greater dissimilarity reflected values closer to 1). We reduced this 40 × 40 RDM, averaging within and across category cells in the same manner as above to yield a 4 × 4 test RDM. The F1–F2 2D distance model was significantly correlated with the vocal tract average model (Spearman *ρ*: 0.94, *P* < 0.005; Kendall *τ*: 0.87, *P* < 0.02), but was not correlated with the stimulus PSD model (Spearman *ρ* and Kendall *τ* both <−0.2, *P* > 0.5). These correlations indicate that although the F1–F2 2D distance model was derived from the stimuli, its representational pattern was distinct from the stimulus PSD model, which included broader spectral content. The high correlation between the F1–F2 2D distance model and the vocal tract model indicates good correspondence in the representation of category-level information based on acoustical cues that derive from articulator position, and from articulator position during imitation of these sounds.

#### RSA Searchlight Analyses

We conducted searchlights on fMRI t-maps. We elected a priori to perform searchlights in regions critical to speech imitation and speech ST. ROIs were defined based on: (1) regions active for listen preimitate, across all vowels (All listen preimitate > rest) and (2) regions active during imitation, across all vowels (All imitation > rest). To limit estimation bias for each ROI contrast, we calculated Jackknifed partial estimates of voxel-wise activation, and subtracted the voxel-wise mean partial estimate from voxel-wise activation for the full cohort. These Jackknifed ROIs were thresholded liberally at whole-brain level, providing coverage of bilateral sensorimotor cortex and anterior cerebellum (imitation; **P** < 0.005, uncorrected), and bilateral sensorimotor cortex, superior temporal gyri and sulci, cerebellum, hippocampus and subcortical nuclei (listen preimitation; *P* < 0.001, uncorrected) (see Fig. 1*d*). Total voxel counts in the ROI volumes were: listen preimitation—12 699; imitation—3112.

Analyses were performed in the ROIs separately, within spherical searchlights (radii: 4.5 mm; ~30 resampled voxels). The 4.5 mm searchlight radius is in line with Kriegeskorte et al. (2006), who showed that searchlight radii of ~4 mm yielded the most optimal performance for unsmoothed data, and for smoothed data with good contrast-to-noise ratio (i.e., 0.3–0.4). In each searchlight, Spearman correlations were used to compare the test 4 × 4 RDM model to the fMRI t-map 4 × 4 RDM (i.e., built by cross-correlating the *t*-values over all voxels in that sphere on a condition-wise basis, expressed as correlation distances) (Fig. 1*d*). During the searchlight procedure, each voxel in the ROIs iteratively served as the center of the sphere. At ROI edges, the searchlight volume was smaller/asymmetric and restricted to voxels that fell within the ROI bounds (i.e., the sphere was centered on a voxel at the ROI mask edge, where the sphere itself was “masked” by the ROI boundary, so that the “spherical” volume was constrained to the voxels within the ROI). In any one sphere, the Spearman correlation between the fMRI t-map and test model RDMs was recorded and reported at the central voxel in that sphere. Second-level group statistics were performed on the resulting voxel-wise Spearman correlation maps using one-sided Wilcoxon signed-rank tests (i.e., testing for positive Spearman correlations only, since negative correlations were not of interest).

We predicted that neural representations reflecting the vocal tract model patterns would emerge at a neural level for both speech imitation and ST; however, we predicted that neural representations reflecting the stimulus (i.e., PSD and F1–F2 2D distance) models would be less likely to emerge during imitation (cf., Cheung et al. 2016). Thus, separate searchlight analyses were performed for ST fMRI data, using the vocal tract, stimulus PSD and F1–F2 2D distance models; the imitation fMRI searchlight analysis was conducted with the vocal tract model only.

## Results

We explored the functional brain basis of speech ST and imitation using a speech production paradigm including rtMRI of the vocal tract and fMRI of the brain. Specifically, we aimed to combine both sources of data, to test the representational basis of vocal tract behavior during ST and imitation.

### Articulatory Training—rtMRI Lip Dynamics

To probe whether training led to lip protrusion for nonnative vowels, we measured lip dynamics using rtMRI while subjects produced trained/untrained and native/nonnative vowels. We tracked the × pixel co-ordinate position of the lips on every trial. For each subject, we calculated the difference in mean lip × pixel co-ordinate between the native and nonnative vowels (i.e., /i/ minus /y/, /a/ minus /ɶ/). Difference scores greater than 0 demonstrated that the nonnative articulatory dynamics had been acquired; i.e., that the lips were protruded for nonnative vowels relative to the corresponding native vowels.

**Figure 2.**
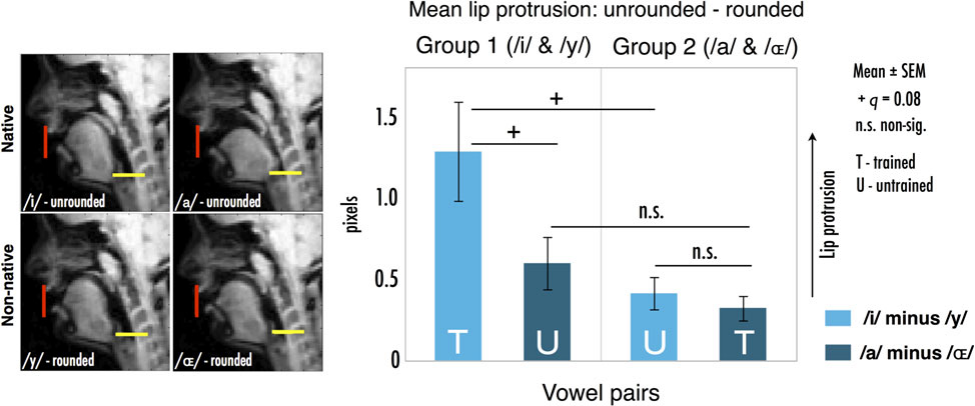
Left: Example lip (red) position traces as measured from rtMRI images in a single subject. Right: Lip protrusion difference metrics per group (unrounded – rounded lip × co-ordinate). Positive values indicate relatively greater protrusion for the rounded than unrounded vowel; note that all means are significantly greater than 0 (all *P* < 0.005; see Results). See Results for description of statistical interaction.

As expected, both groups achieved significant extents of lip protrusion for the trained nonnative vowels (Fig. 2) (planned one-sample *t*-tests of difference [unrounded – rounded] vs. 0: groups 1 and 2 both *t* > 4.0, **P** < 0.005). Moreover, we found that training on imitating one nonnative vowel extended to the nonnative vowel that subjects had not practised before scanning. Thus, on average, subjects also protruded their lips successfully for the untrained nonnative vowel (planned one-sample tests of untrained diff. [unrounded – rounded] vs. 0: groups 1 and 2 both *t* > 3.6, *P* < 0.005).

Owing to differences in training conditions and open versus close vowel dynamics (i.e., jaw position) between the 2 vowel pairs, we found that lip protrusion varied due to vowel and training group. There was a marginal interaction of these factors [*F*(1,22) = 3.83, *P* = 0.063, $\eta_{p}^{2}=0.148$], and significant main effects of each factor [vowels/training group: *F*(1,22) = 6.5/7.4, *P* = 0.018/0.012, $\eta_{p}^{2}=0.228/0.253$]. Due to a training advantage and the greater extent of lip protrusion anatomically possible for the close /y/ vowel, group 1 protruded their lips marginally more for /y/ (trained) than /ɶ/ (untrained) [*t*(11) = 2.38, *q* = 0.08]. However, group 2 showed no such difference between vowels (*q* > 0.3) (Fig. 2). Group 1 also protruded marginally more for /y/ than did group 2 [*t*(13.3) = 2.73, *q* = 0.08]; this was expected, since group 2 did not train on /y/ (Fig. 2) (all tests false discovery rate [FDR] corrected). The lesser protrusion overall for the open /ɶ/ likely reflects the lip dynamics that were feasible for this vowel (limited by requisite lowering of the jaw); nevertheless, training on /ɶ/ was still followed by lip protrusion for the untrained /y/.

### Univariate fMRI Analyses

We targeted the listen preimitation and imitation portions of the speech production trials with separate flexible factorial 2 × 2 ANOVAs in SPM (factors: trained/untrained, native/nonnative); this let us explore activation across conditions for prearticulatory ST and subsequent imitation, respectively.

**Figure 3.**
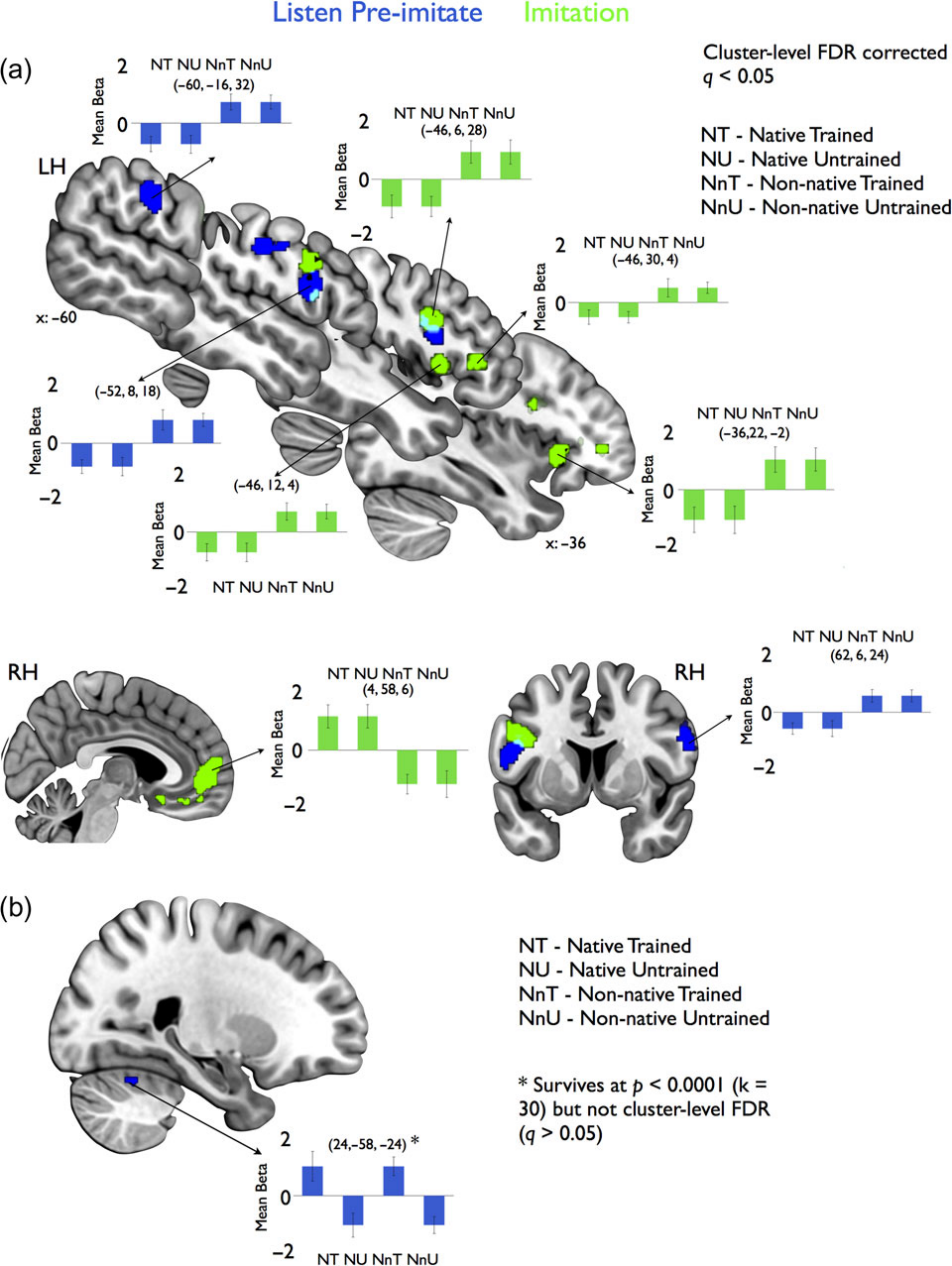
Univariate 2 × 2 ANOVA results (factors: training, native/nonnative) for ST (blue) and imitation (green) fMRI data. (*a*) Native/nonnative 2 × 2 main effect results for ST (blue) and imitation (green), significant at cluster-corrected FDR level (*q* < 0.05). Bar plots display mean beta parameter estimates (adjusted response) for cluster peak voxels (peak co-ordinates in parentheses). Conditions: NT, native trained; NU, native untrained; NnT, nonnative trained; NnU, nonnative untrained. (*b*) Training main effect results for ST (blue), significant at **P** < 0.0001 (*k* = 30) (did not survive at cluster-level FDR for voxel-height threshold of *P* < 0.0015, *k* = 50; *q* > 0.05).

Modeling the main effect of native versus nonnative vowel status, there was significant activation (**P** < 0.0015, *k* = 50, achieving cluster-level FDR *q* < 0.05) in inferior frontal and parietal speech regions for ST, together with activation in inferior frontal regions during vowel imitation (Fig. 3*a*). Activation was distributed for ST (blue clusters, Fig. 3*a*), and greater for nonnative than for native vowels in all of these regions; activated regions included left inferior frontal gyrus (IFG; BA 44), left somatosensory cortex, and right ventrolateral motor cortex. For vowel imitation, activation (green clusters, Fig. 3*a*) was significantly greater for nonnative than native vowels in left anterior insula, left IFG (BA 44 & 45), and left lateral premotor cortex. Significantly greater activation for native than nonnative vowel imitation occurred at right medial prefrontal cortex (Fig. 3*a*, bottom). Although not hypothesized a priori, this effect may reflect differential recruitment of nonspeech attentional or default networks, as a function of imitation complexity (i.e., greater recruitment in the less complex native condition; Geranmayeh et al. 2014).

In exploring the main effect of training, activation during ST for trained vowels was greater than for untrained vowels at right anterior cerebellum (Fig. 3*b*). This suggests that regions involved in motor performance were engaged during the preimitation preparatory ST period, in the absence of any overt articulation. While this activation was significant at a voxel-height threshold of *P* < 0.0001 (*k* = 30), it did not survive with cluster-level FDR correction (at *P* < 0.0015, *k* = 50; *q* > 0.05). No regions showed significant effects of training on activation during vowel imitation.

Finally, neither 2 × 2 analysis yielded evidence of clusters that showed significant two-way interactions.

### Representational Similarity Analysis

To probe representation of vowel ST and imitation in speech sensorimotor regions, we built test models of vowel production derived from images of the vocal tract. Further, we constructed stimulus test models derived from the spectral properties of stimuli, and the inter-stimulus distances in formant space. In a series of analyses, we compared RDM patterns derived from vocal tract images of vowel articulation to patterns of fMRI activation in searchlights within functionally defined speech imitation and speech ST ROIs. We combined both training groups in RSA second-level analyses, since we aimed to probe ST and production effects common to all subjects.

### RSA 1: Sensorimotor Transformation

**Figure 4.**
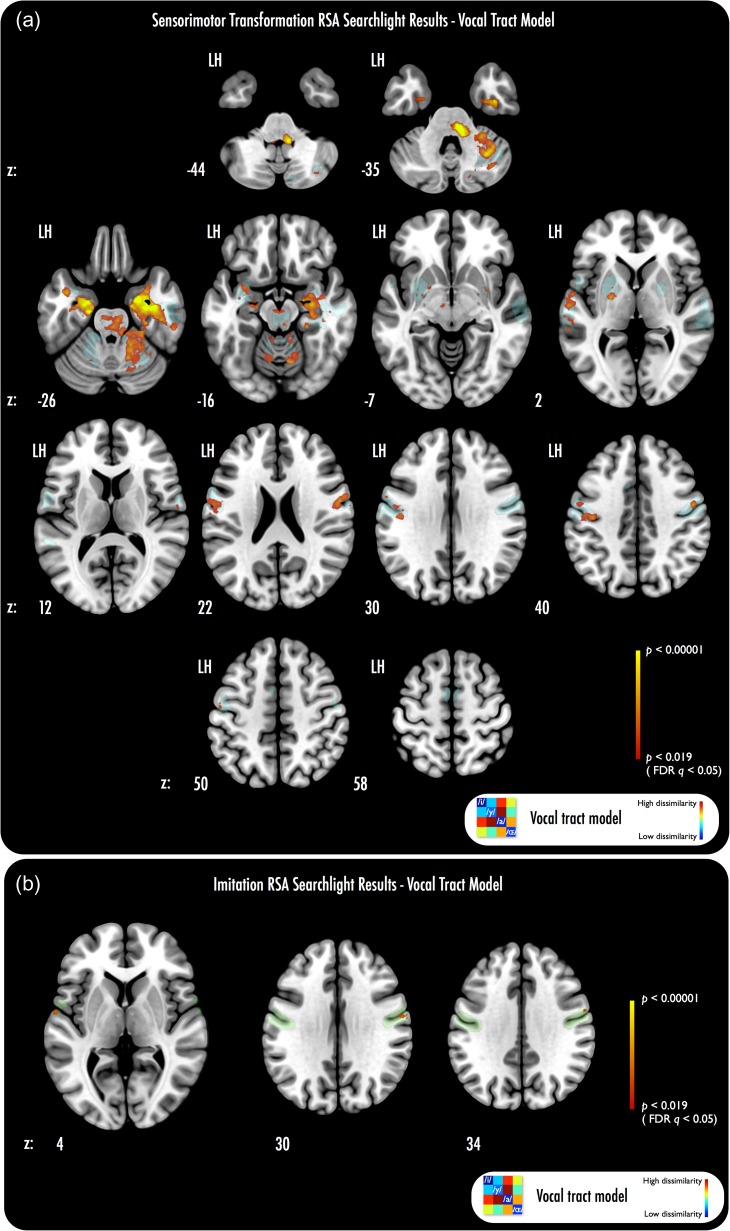
RSA searchlight results. (*a*) Vocal tract group average RDM model pattern correlates with fMRI activation patterns in bilateral somatomotor, left superior temporal, bilateral medial temporal and right cerebellar regions for ST. The stimulus acoustic-derived RDM pattern did not correlate robustly with fMRI t-map RDMs; tests of the correlation coefficients from both analyses showed significantly more robust correlations for the vocal tract model than the stimulus model (note that this overlapped with all voxels where significant vocal tract model and fMRI t-map correlations emerged; *q* < 0.05, FDR-corrected). (*b*) Vocal tract group average model correlates nonrobustly with fMRI activation patterns for imitation in left ventral M1/lateral Heschl's gyrus, and right lateral somatomotor cortex. Transparent underlays in (*a*) and (*b*) show the boundaries of the searchlight ROI volume—blue: ST ROI; green: imitation ROI. Scale bar minimum in (*a*) shows the equivalent uncorrected threshold at which voxel-height FDR correction (*q* < 0.05) is achieved; for consistency, the same scale bar range is used in (*b*), but note that (*b*) correlations are nonsignificant with FDR correction (*q* > 0.05).

All models were tested within the speech ST ROI (see RSA Searchlight Analyses). As predicted, we found that the group average vocal tract RDM was significantly correlated with fMRI activation patterns during ST (within-ROI peak-level FDR-corrected *q* < 0.05; Fig. 4*a*). Table 1 presents peak co-ordinates in Montreal Neurological Institute (MNI) space for the searchlight correlations. Regions that yielded significant correlations included: bilateral somatomotor cortex, hippocampus and parahippocampal gyrus, cerebellum (lobule V/VI, Cru I), left superior temporal lobe, and bilateral putamen (Fig. 4*a*). Correlations with the vocal tract model in precentral gyrus and post-central gyrus were revealed as a series of clusters across each hemisphere, with foci at ventrolateral precentral gyrus extending across the central sulcus onto post-central gyrus; further clusters were observed in more dorsal precentral gyrus locations bilaterally, and at subcentral gyrus bilaterally. Correlations across left superior temporal lobe occurred as 2 major clusters, one extending from lateral Heschl's gyrus to anterior STG and STS, and the other located at posterior STS. Correlations in subcortical structures included a cluster that covered much of left putamen, with a further neighboring cluster at left globus pallidus. A smaller homolog of the left putamen cluster also manifested at right putamen. Correlations across medial, anteromedial and ventral temporal lobe (MTL, aMTL, VTL) were extensive, covering much of the anterior hippocampus bilaterally, in addition to parahippocampal gyrus, right collateral sulcus, and left aMTL (proximal to the ventral boundary with circular sulcus). Correlations were also extensive within the cerebellum, covering much of lobules V/VI bilaterally, in addition to Crus I in the right cerebellum. A large cluster also emerged within the pons, lateralised to the right of the midline.

**Table 1:** Peak voxel co-ordinates (MNI space) and locations from RSA searchlights

Analysis	Cluster	*x*	*y*	*z*	*z*-Score	Voxel count over clusters (*P* < 0.005)	Voxel count over clusters (FDR *q* < 0.05)
Listen preimitate—group average vocal tract RDM	LH Central Sulcus	−42	−20	38	3.83		
	LH Precentral Gyrus (i)	−50	−12	44	3.29		
	LH Precentral Gyrus (ii)	−58	2	34	2.81		
	LH Lateral Heschl's Gyrus	−60	−12	4	3.48		
	LH Lateral STG	−66	−16	−2	3.78		
	LH STS	−62	−22	−2	3.48		
	LH Putamen	−26	−4	−2	3.48		
	LH Globus Pallidus	−20	−6	−2	3.22		
	LH Anterior Hippocampus	−22	−14	−20	4.16		
	LH Anterior Hippocampus/Amygdala	−22	−8	−26	4.85		
	LH Entorhinal Cortex	−20	−16	−26	4.85		
	LH Anteromedial Temporal Lobe	−40	2	−22	3.78	2875	4874
	LH Anterior Cerebellum (lobule V/VI)	−12	−58	−20	3.54		
	RH Precentral Gyrus (dorsal)	50	−6	42	3.48		
	RH Precentral Gyrus (ventral)	56	−2	22	3.48		
	RH Putamen	30	0	−4	2.45		
	RH Anterior Hippocampus	32	−12	−24	4.47		
	RH Anterior Hippocampus/Amygdala	24	−6	−26	5.16		
	RH Collateral Sulcus	42	−12	−30	5.16		
	RH Pons	8	−32	−32	5.07		
	RH Anterior Cerebellum (lobule V/VI) (i)	30	−38	−32	4.05		
	RH Anterior Cerebellum (lobule V/VI) (ii)	18	−44	−28	3.89		
	RH Cerebellum (Crus I)	34	−56	−34	4.26		
Imitate—group average vocal tract RDM	LH Lateral Heschls/Ventral M1	−60	−4	4	2.88		
	RH Lateral M1	60	0	34	3.02	7	0
	RH Lateral S1	60	−6	30	3.02		

Exploring these findings in more detail, we ran searchlights at the single-subject level, using each subject's average 4 × 4 vocal tract RDM as the test model in a searchlight of their own ST fMRI data (see Supplementary Fig. 2). Group statistics were then calculated across the resulting subject-wise Spearman correlation maps. The analysis showed extensive evidence of significant voxels that overlapped closely with the results noted above for the cohort-average 4 × 4 vocal tract RDM (see Supplementary Fig. 1). We tested the difference in significance of the Spearman correlation maps derived from the average and the subject-specific vocal tract models (using voxel-wise Wilcoxon signed-rank tests); we found no evidence of robust differences in voxel-wise correlations (FDR-corrected *q* > 0.05), and observed only small clusters at left anteromedial temporal lobe and right collateral sulcus that showed stronger correlations for the subject-specific than the group average models (*P* < 0.001, uncorrected; data not shown).

Thus, we found that test representational patterns built from vocal tract images of articulation across all 4 vowel categories were significantly correlated with fMRI activation patterns during ST that “preceded” the imitation of the vowels. Further, similar results were observed both when using group averaged and subject-specific vocal tract RDMs in searchlight analyses of ST data.

**Figure 5.**
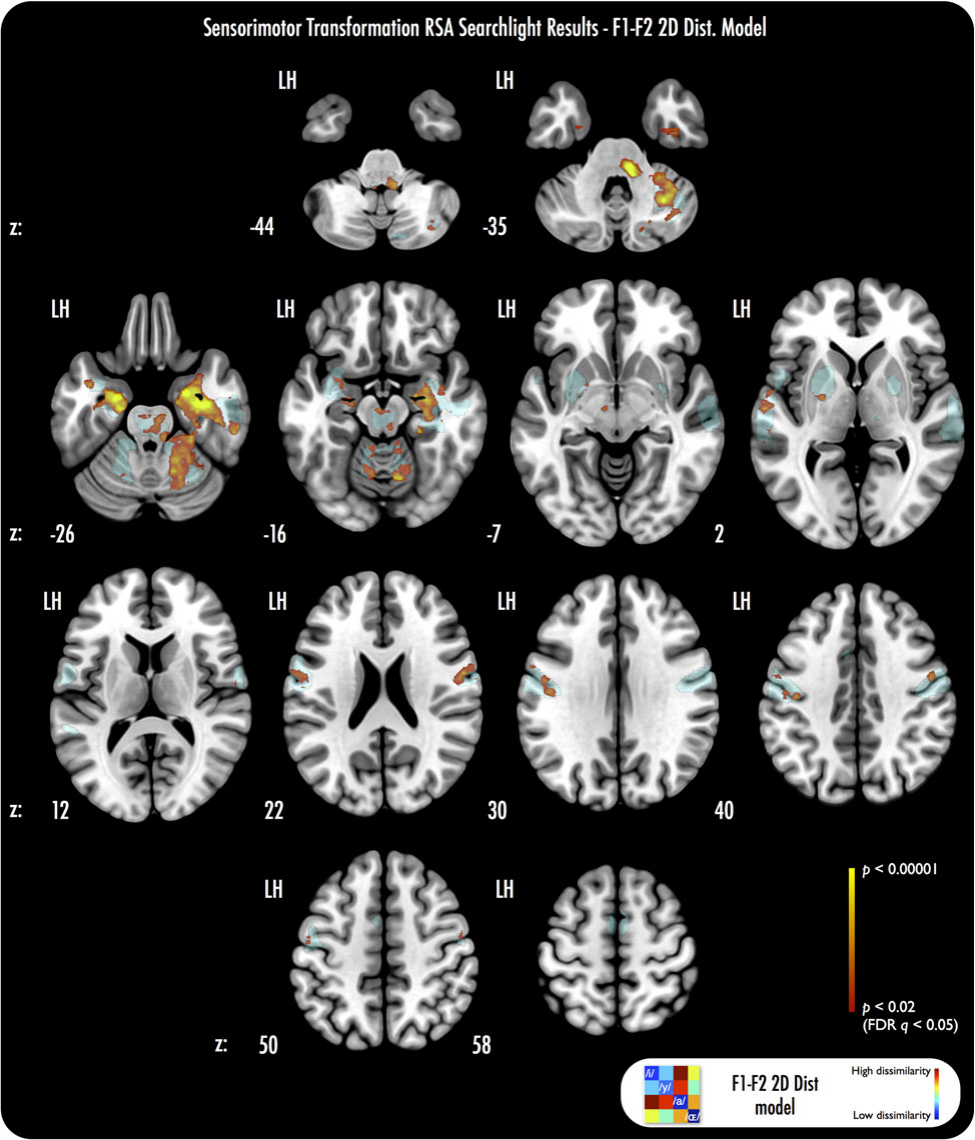
RSA searchlight results using F1–F2 2D Euclidean distance RDM test model. The F1–F2 2D Euclidean distance model reveals correlations that overlap most of the regions that manifested significant searchlight correlations for the group average vocal tract model (see Fig. 4). Voxel-wise Wilcoxon signed-rank tests of the correlation maps derived from the vocal tract average model and the correlation maps from the F1–F2 2D Euclidean distance model, did not reveal any significant differences in robustness of the correlations across the 2 analyses (all FDR *q* > 0.05). All other parameters as per Figure 4.

Probing the representational bases of the ST data further, we used the stimulus PSD and F1–F2 2D Euclidean distance models as inputs to searchlights of preimitation listening fMRI data within the ST ROI. We found no evidence of significant correlations in the ST ROI between the stimulus PSD model and fMRI activation patterns (no searchlights survived at FDR-corrected *q* < 0.05, nor **P** < 0.005, uncorrected). However, the F1–F2 2D distance model did yield robust correlations (FDR-corrected *q* < 0.05) with ST fMRI activation patterns (see Fig. 5), across many of the same regions that showed significant correlations for the vocal tract model searchlights of ST data. We tested the difference between the correlation maps for the average vocal tract, stimulus PSD, and F1–F2 2D distance models in the ST ROI (with voxel-wise Wilcoxon signed-rank tests). We found that the average vocal tract model yielded significantly more robust correlations with the ST fMRI activation patterns than did the stimulus PSD model, at all voxels that had shown significant correlations with the vocal tract model in the first searchlight analysis (FDR-corrected *q* < 0.05). Similarly, we found that the F1–F2 2D distance model revealed significantly more robust correlations with ST activation patterns than did the stimulus PSD model, across all voxels that had shown significant correlations with the F1–F2 2D distance model in the initial analysis (FDR-corrected *q* < 0.05). We found no evidence of any significant differences in robustness of correlations between the vocal tract average model and the F1–F2 2D distance model (all FDR *q* > 0.05).

In summary, we found that test representational patterns built from vocal tract images of vowel articulation and from the distances between the stimuli in vowel formant space were significantly correlated with fMRI data obtained during ST that preceded the imitation of these vowels. Further, the vocal tract and F1–F2 2D distance RDM test models yielded more robust correlations with the ST fMRI data than did the speech stimulus PSD model.

### RSA 2: Speech Imitation

We next explored the representational basis of speech imitation. We predicted that the vocal tract RDM model would reveal representational patterns in the imitation fMRI data, for searchlights within the speech imitation ROI.

We found very limited evidence of correlations between the test vocal tract and searchlight RDM patterns at the group level. Several small peaks manifested within our speech imitation ROI at right lateral precentral gyrus and post-central gyrus; a small peak also emerged at the boundary between left ventral precentral gyrus and left lateral Heschl's gyrus (Fig. 4*b*). These observed peaks were significant at an uncorrected threshold of **P** < 0.005, but did not survive with FDR correction (*q* > 0.05).

Hence, we found that the vocal tract RDM test model revealed a quite limited set of regions where searchlight correlations with fMRI activation patterns for imitation survived at uncorrected thresholds; those searchlight correlations did not survive correction for multiple comparisons.

### RSA 3: Speech Perception

While the primary focus of our present RSA analyses was on ST and speech imitation, we also considered whether representation of the acoustically based and vocal tract image-based RDMs would emerge during passive listening (i.e., during the listen only trials that occurred pseudorandomly amongst the ST/imitation trials). We defined an ROI for RSA searchlight analyses, based on regions that were active in the univariate contrast of all listen only > rest (using the same Jackknifing procedure as for the ST and imitation ROIs; the Jackknifed mask was thresholded liberally at **P** < 0.05 uncorrected, providing coverage of superior temporal gyrus and sulcus [total mask voxel count = 941]). In separate searchlight analyses, we used the group average vocal tract RDM, the stimulus PSD RDM, and the stimulus F1–F2 2D Euclidean distance RDM as test models which we compared with the patterns amongst the condition-wise listen only t-maps from our fMRI analyses. We found that only the stimulus PSD RDM revealed any evidence of searchlight correlations significant at *P* < 0.005 (uncorrected). These manifested as a small cluster at left anterior STG; however, the correlations were nonrobust to FDR correction across the ROI voxels. We include the results from the stimulus PSD searchlight analyses for the interested reader as Supplementary Fig. 3, at an uncorrected threshold; however, we note that these results did not survive correction for multiple comparisons and should be interpreted cautiously.

## Discussion

Here, we demonstrate that the neural representations of speech ST can be revealed by images of the speaking vocal tract, and by the acoustic correlates of vowel articulation. Our results shed light on the extensive functional brain networks involved in preparing to articulate imitations of vowels that varied in familiarity; these results unveil the topography of regions involved in ST for vowel categories differing in their articulatory and acoustical properties, over and above results obtained using more traditional univariate BOLD analyses.

Using a speech production paradigm, we trained monolingual adults to imitate native and nonnative vowel targets. Central to our study was probing training outcomes directly from the motor effectors used for speech, via real-time vocal tract MR imaging. We found that participants were successful in acquiring the lip protrusion dynamics for trained nonnative vowels, and also that they extended these dynamics to an untrained nonnative vowel during scanning. While vocal tract dynamics have previously been measured for vowel articulation (e.g., tongue movements: Niebergall et al. 2013), we show here that labial tissue metrics allow measurement of articulatory performance for nonnative speech. Speech imitation studies have demonstrated that practice leads to reduced acoustic distance between target and imitation formants (Kartushina et al. 2015), but no study yet has shown the acquisition of specific nonnative articulatory dynamics with rtMRI. We thus present the first MRI data collected directly from the vocal tract to show successful articulatory learning for nonnative speech.

Having determined behaviorally that nonnative vowels were produced with the requisite articulatory dynamics, functional MRI allowed us to explore neural correlates of both speech ST and imitation. Univariate results showed effects of nonnativeness and training during ST, and of nonnativeness during overt imitation. In line with existing literature (Moser et al. 2009; Golestani and Zatorre 2004; Perani et al. 2003; Simmonds et al. 2011), we found increased activation in left anterior insula and left IFG during nonnative vowel imitation; this likely reflects the taxing of phonological and articulatory processes by these vowels (see Riecker et al. 2008). Activation was also increased in left post-central and right precentral gyri during ST for nonnative vowels. This result extends previous findings of greater activation in premotor regions during nonnative speech production (Simmonds et al. 2011) to include sensorimotor cortex during ST; this early sensorimotor cortex engagement during ST may have served to buttress the vowel articulations that followed. Activation was also increased in right anterior cerebellum for trained vowels during ST. Cerebellar activation has previously been found during covert speech: McGettigan et al. (2011) showed increased right-lateralised cerebellar activation during covert pseudoword rehearsal when contrasting 4- versus 2-syllable items. Further, “decreases” in right-lateralised cerebellar activation have also been found across repeated instances of covert repetition of novel pseudowords (Rauschecker et al. 2008). Modulation of cerebellar activation has additionally been reported during the initial learning of nonspeech motor sequences (e.g., Doyon et al. 2002). Here, the increased right cerebellar activation we found during ST may have reflected an anticipatory or preparatory recruitment of speech motor network subregions for the more familiar trained vowels.

Recent multivariate analyses of speech processing have indicated an array of regions involved in perception, ST and production of speech. Data now suggest that representations span a network of somatomotor, superior temporal and inferior frontal areas across perceptual, preparatory and articulatory stages of speech (Cogan et al. 2014; Du et al. 2014; Evans et al. 2014). Such stages reflect phoneme selection, transformation to motor targets, articulation, and relay of motor efference copies to sensory regions (e.g., Guenther 2006; Niziolek et al. 2013; Simmonds et al. 2014b). Hierarchies of abstraction appear within these networks during perception, such that the spectra of complex acoustic signals are represented in belt and parabelt auditory regions (Davis and Johnsrude 2003; Joanisse and DeSouza 2014), whilst abstract categorical or phonemic dimensions of speech are also represented in somatomotor areas (Evans and Davis 2015; see also Pülvermüller et al. 2006). Some studies have further identified cortical representation of phonetic features such as place and manner of articulation, during production (Bouchard et al. 2013; Cheung et al. 2016) and perception (Arsenault and Buchsbaum 2015; Correia et al. 2015; though see also Mesgarani et al. 2014; Cheung et al. 2016) of speech. Yet, to date, very little work that has explored representation of speech has specifically sought to relate the distinct sources of information from vocal tract dynamics and speech acoustics to their underlying neural representations, for both ST and imitation.

Multivariate RSA combining fMRI, rtMRI and formant distance data from our study enabled the identification of neural representations during ST. Thus, we show that the relational patterns amongst speech categories that reflect the distinct positioning of the articulators, and/or the corresponding distances amongst vowels in acoustic space, appear to be preserved at a neural level during ST. An important caveat regarding the present analyses is that our test models built from images of the speaking vocal tract, and from the distances between vowels in formant space, were highly correlated. Thus, while we observed no evidence of significant differences in the searchlight results derived from the 2 models, it is not possible to conclude that the models derived from these different data sources (i.e., MR vs. acoustics) reflected distinct representations that co-occurred during speech ST. Indeed, given that F1 and F2 vary as a function of tongue height and frontness, respectively, the models here share a good degree of commonality in terms of the broader behavioral source of the category separation reflected in each.

Nevertheless, our finding that representational patterns for different vocal tract configurations and/or vowel category acoustic distances are represented within ST networks builds on multivariate accounts of hierarchical representations in speech regions. Furthering previous multivariate accounts of ST (Cogan et al. 2014), our results using vocal tract/F1–F2 2D distance models revealed an extensive representational topography of speech targets during ST that included somatomotor, temporal, hippocampal, cerebellar, and subcortical regions (putamen and globus pallidus). Importantly, we found that correlations between ST activations and our stimulus PSD searchlight model were much less robust than those observed for the vocal tract models, or for the F1–F2 2D distance model (as noted above, the latter reflecting the acoustic correlates of articulator position—F1 relates inversely to tongue height, whereas F2 relates directly to tongue frontness). This affords the first RSA evidence that activation patterns during ST more keenly reflect the representation of target articulator positions—and/or the acoustical features tied to these—that are essential to speech (see Cogan et al. 2014). Moreover, we showed that ST activation patterns were better fit by these properties, and diverged from test patterns based on representational distances derived from the raw spectral properties of vowel categories. Taken together, our results suggest a common representation of the categorical dimensionality of vowels during ST, which corresponds well with patterns indicative of the position of the articulators when vowels are spoken, and/or patterns reflecting the distances amongst the key acoustical determinants of vowel category identity (i.e., formants). In addition, we extend the results of Evans and Davis (2015), who reported representation of syllable identity in left lateral somatomotor cortex during passive perception that overlapped with regions active during speech production (as observed through their univariate analyses). Our findings suggest that active ST also involves robust representation of distinctions between speech categories based on differences in vocal tract position and/or related acoustical category distances, within similar lateral somatomotor regions.

A further advantage of the present searchlight approach was the potential to probe representations across the full extent of voxels that were active during ST (as determined by univariate subtraction). Many existing multivariate accounts of the representational basis of ST (and indeed, imitation) have largely been confined to analyses of activity on the lateral cortical surface; this has reflected the coverage achievable with electrocorticography methods (Cogan et al. 2014; see also Bouchard et al. 2013), or a methodological choice to use surface-only analyses in fMRI (Correia et al. 2015; Markiewicz and Bohland 2016). Our approach enabled us to run searchlights within the full extent of regions involved in ST, and implicated bilateral cerebellum, striatum, and hippocampi within the representational network of speech ST. Indeed, the clusters we observed across cerebellum (particularly in the right cerebellar hemisphere) and bilateral putamen are compatible with the involvement of these regions in speech articulatory performance (Riecker et al. 2008; Segawa et al. 2013; Simmonds et al. 2014a). Moreover, our findings now point toward the recruitment of these cerebellar and subcortical networks in representing categorical distinctions based on articulator position and/or acoustic distances, at the earlier prearticulatory stage of ST.

Of particular interest was our finding that MTL regions including the hippocampus also represented the vocal tract/F1–F2 2D distance model patterns during ST. While hippocampal activation has been found to be modulated by familiarity and success of recall for novel lexical items (Davis et al. 2009), we believe ours to be the first multivariate results to show correlations between MTL activation during ST and models specifying differences in categorical relationships based on articulator position or acoustic distance. Previous fMRI studies have shown that the online maintenance of sensory information during a working memory task activates anterior hippocampus (e.g., Ranganath and D'Esposito 2001). Moreover, multivariate fMRI investigations have further shown that subregions within MTL vary in the extent to which they code selectively for category-specific stimuli, with parahippocampal gyrus responding selectively to visual category identity (Diana et al. 2008). A recent multivariate analysis of auditory fMRI data further found that hippocampus activation showed selectivity for novel tone cloud categories that repeated across trials versus tone clouds that did not, during an active repetition-monitoring task (Kumar et al. 2014). Taken together, these studies suggest that at least some medial temporal structures appear to preserve information concerning categorical relations, appear to do so across several modalities, and are involved where there is some active need to maintain information online. We suggest that it is therefore possible that the patterns of dissimilarity between articulator positions and/or acoustic category distances that reflect distinct vowel categories are further represented within MTL during prearticulatory ST.

Counter to our prediction, we found only modest correlations of the group-defined vocal tract model with speech imitation activation patterns; these correlations were nonrobust to correction for multiple comparisons. Challenges in probing speech somatomotor representations with fMRI concern the granularity at which representations are expected to emerge, together with considerations of the analysis of the BOLD signal. For instance, recent electrocorticography investigations have shown consistent evidence of a broadly somatotopic ordering of activity for speech phone articulation across the ventrolateral half of somatomotor cortex, with anterior articulators (e.g., lips) mapped dorsal to more posterior articulators (e.g., tongue) (Bouchard et al. 2013; see also Cheung et al. 2016). Further, a recent fMRI study that used phase-encoded analysis methods to map articulator positioning revealed maps similar to those of Bouchard et al. (2013), but found limited evidence of activation differences across conditions when block contrasts of each articulation condition versus rest were used (Carey et al. 2017). One possibility is that our present searchlight analysis did not reveal robust correlations within somatomotor regions due to voxels within the local searchlights reflecting similar extents of activation across the 4 conditions. For instance, a particular set of searchlight voxels in a given somatomotor region could show largely consistent amplitude of the BOLD response across vowel categories, when each condition is contrasted with rest (e.g., Grabski et al. 2012). In such an instance, the resulting RDM pattern emerging from the fMRI t-maps in that searchlight could reflect correspondingly low dissimilarity across all possible condition pairs—this would not have fit with the vocal tract or 2D formant distance model patterns, which showed dissimilarity that varied between vowel category pairs. With respect to ST, it is possible that the convergence of many distinct functional processes (e.g., speech perception, sensory memory, phoneme selection and competitor suppression, speech motor program mapping) may manifest via more condition-specific variation in BOLD signal amplitude at the level of local voxels (as found within a searchlight volume); this may lead to neural patterns that more readily correlate with the condition-wise categorical relationships found in independent RDMs, as generated from vocal tract images or formant distances. In future studies, an approach such as a phase-encoded experimental design could enable us to parcellate the BOLD response in somatomotor regions into maps of articulatory differences between vowel conditions (further to Carey et al. 2017); we note however that such a design was outside of the scope and aims of our present study.

One further issue is that the spatial resolution of our vocal tract images may have been a limiting factor in allowing us to capture more fine-grained facets of articulator somatomotor representations during imitation (see Brown et al. 2008; Meier et al. 2008; see Takai et al. 2010). Improvements in the spatial resolution of our rtMRI data will allow more refined test models to be built, which may offer a better fit to patterns of cortical representation during imitation. One recent study that used images of the lips in combination with direct cortical recordings during speech found that cortical activity could be predicted in one subject from time-varying traces of lip aperture (Bouchard et al. 2016). This suggests promise for future advances in studying articulation in cortex, where high-resolution articulator imaging can be integrated with high-resolution functional imaging data. Sensitivity to fine-grained variations in articulator position might also be improved by modified MR acquisition paradigms allowing for within-trial recording of both vocal tract and brain volumes (see Paine et al. 2011).

In seeking to examine separately the neural correlates of speech ST and imitation, our approach is similar to a recent study of delayed syllable repetition in fMRI (Markiewicz and Bohland 2016). Using multivoxel pattern classification with cortical surface searchlight analyses run on (1) responses to the initial auditory presentation of the speech item and (2) responses to a subsequent visual cue to repeat it, the authors identified regions showing significantly above chance coding of vowel identity (/Ι/, /ε/, and /Λ/), in a range of speech sensorimotor regions. In contrast to our study, they found much stronger evidence for prediction of vowel identity in output-related activations than during responses to the auditory input. There are a number of factors to consider here. Markiewicz and Bohland were studying more complex utterances, and their design mainly probed abstract categorical (i.e., phonemic) representations of vowels in their stimuli (cf., our vocal tract and acoustic stimulus models). Further, speech production was cued 8–9 s after auditory presentation and participants were explicitly instructed to repeat without acoustic imitation; this potentially tapped into more abstract representations of speech than in the current task, which focused on precise imitation. We suggest that future work should more systematically measure the effects of stimulus properties, task demands and event timings on the neural responses to speech, in order to obtain more comprehensive accounts of how these factors interact with the specificity and granularity of speech cortical (and subcortical) representations during ST and imitation.

Our approach using RSA holds translational potential for speech in clinical settings. Following traumatic brain injury or stroke, vocal tract imaging may enable insights into patients’ articulatory difficulties (see Vasquez Miloro et al. 2014); vocal tract images could additionally be compared with fMRI data collected in the same patients during ST or speech, using RSA. This may improve understanding of clinical speech pathology, through quantifying adaptations over the course of speech rehabilitation, and identifying sites for neural interventions in conjunction with behavioral therapy (see Holland et al. 2011).

In summary, we provide the first evidence that images of the speaking vocal tract and acoustic measures of speech category distances can allow neural representations of speech ST to be charted. These insights, allied to direct measures of vowel articulatory dynamics, afford a unique multidimensional account of the mechanisms supporting speech ST.

## Supplementary Material

Supplementary Data

Supplementary Data
