# Supplementary material for: Vocal Tract Images Reveal Neural Representations of Sensorimotor Transformation During Speech Imitation
Source: Cereb Cortex. 2017 Mar 18;27(5):3064–79. doi: 10.1093/cercor/bhx056 (PMC5939209; doi:10.1093/cercor/bhx056)
Supplement: Supplementary Data [file Carey_et_al_CerebralCortex_Supplementary_Legends.doc]

**Supplementary Figure 1**

RSA searchlight results using individual subject vocal tract RDM test models. Each subject’s own 4 x 4 vocal tract image-derived RDM was used in a searchlight analysis of the subject’s own fMRI t-maps (see Supplementary Fig. 2 for subject-wise vocal tract image-derived RDMs). We then calculated group statistics over the Spearman correlation maps that were calculated per subject. Results showed searchlight correlations that were co-extensive with the group average vocal tract RDM results (see Figure 4). Voxel-wise Wilcoxon signed rank tests comparing the results from the group average and individual subject vocal tract RDMs did not reveal any robust differences in correlation maps between the two analyses (all FDR *q* > 0.05).

**Supplementary Figure 2**

Individual subject vocal tract image-derived RDMs. Each panel presents the subject-specific 4 x 4 average model used as input to searchlight analyses of that subject’s own fMRI t-maps. Each 4 x 4 model was derived from images of a particular participant’s vocal tract as they spoke each of the vowels across the rtMRI blocks in the experiment (see Methods for details of vocal tract RDM construction).

**Supplementary Figure 3**

RSA Searchlight results using the stimulus PSD model in searchlight analyses of passive listening trials. (a) Searchlight results show correlations between listen only fMRI t-maps and stimulus PSD RDM, that survive at *p* < 0.005, uncorrected (cluster highlighted with mauve circle). Note that searchlight results with stimulus PSD model did not survive correction for multiple comparisons (FDR *q* > 0.05). (b) Speech perception ROI used to constrain searchlight analyses of listen only fMRI data.
