# Supplementary material for: Vocal Tract Images Reveal Neural Representations of Sensorimotor Transformation During Speech Imitation
Source: Cereb Cortex. 2017 Mar 18;27(5):3064–79. doi: 10.1093/cercor/bhx056 (PMC5939209; doi:10.1093/cercor/bhx056)
Supplement: Supplementary Data [file Carey_et_al_Vocal_tract_images_reveal_representations_of_speech_sensorimotor_transformation_during_speech_imitation_Suppl_Figures_Revised.pdf]

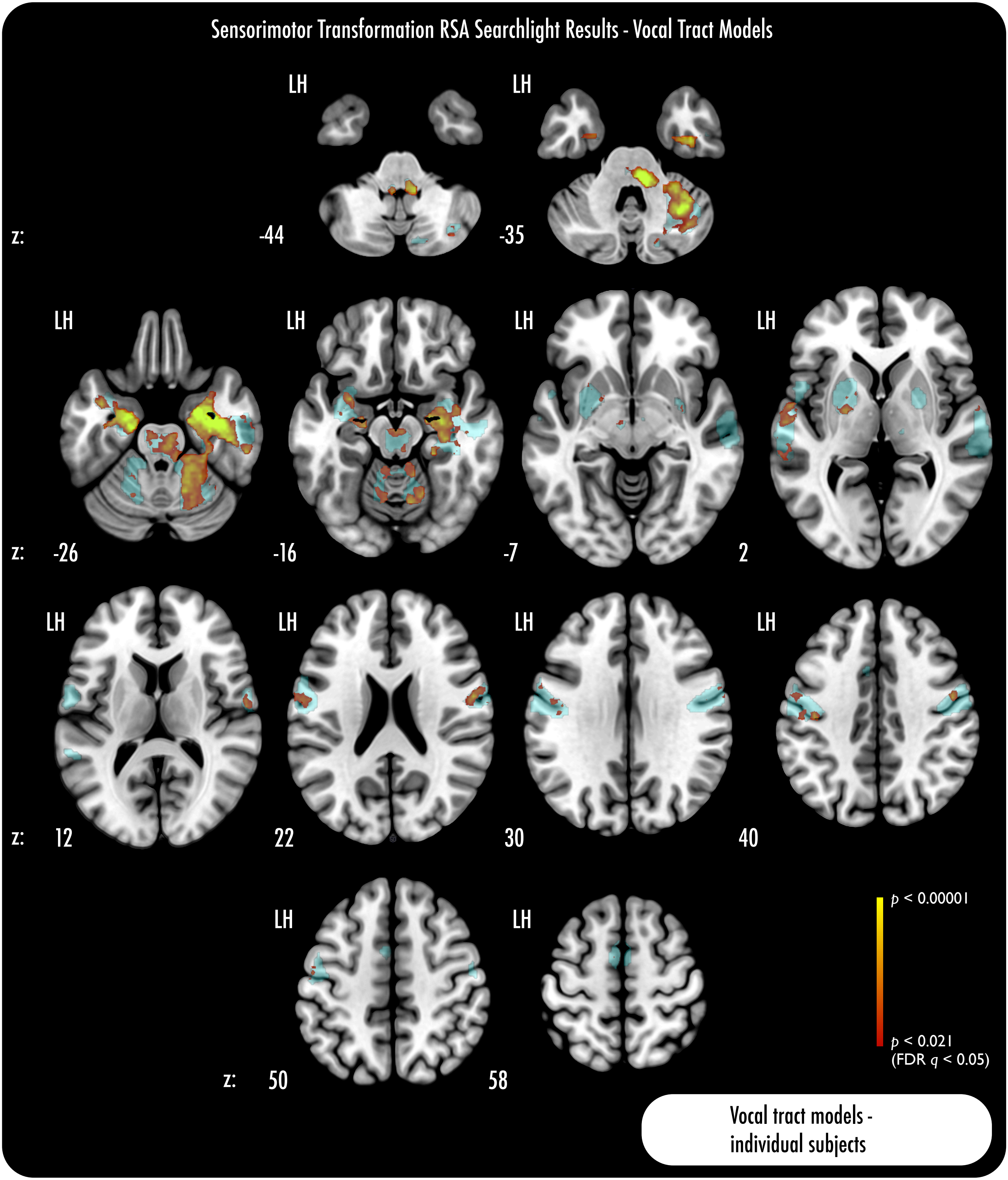
Sensorimotor Transformation RSA Searchlight Results - Vocal Tract Models
LH
z:
-44
-35
-26
-16
-7
2
12
22
30
40
50
58
p < 0.00001
p < 0.021
(FDR q < 0.05)
Vocal tract models - individual subjects

Individual Subject Vocal Tract RDMs

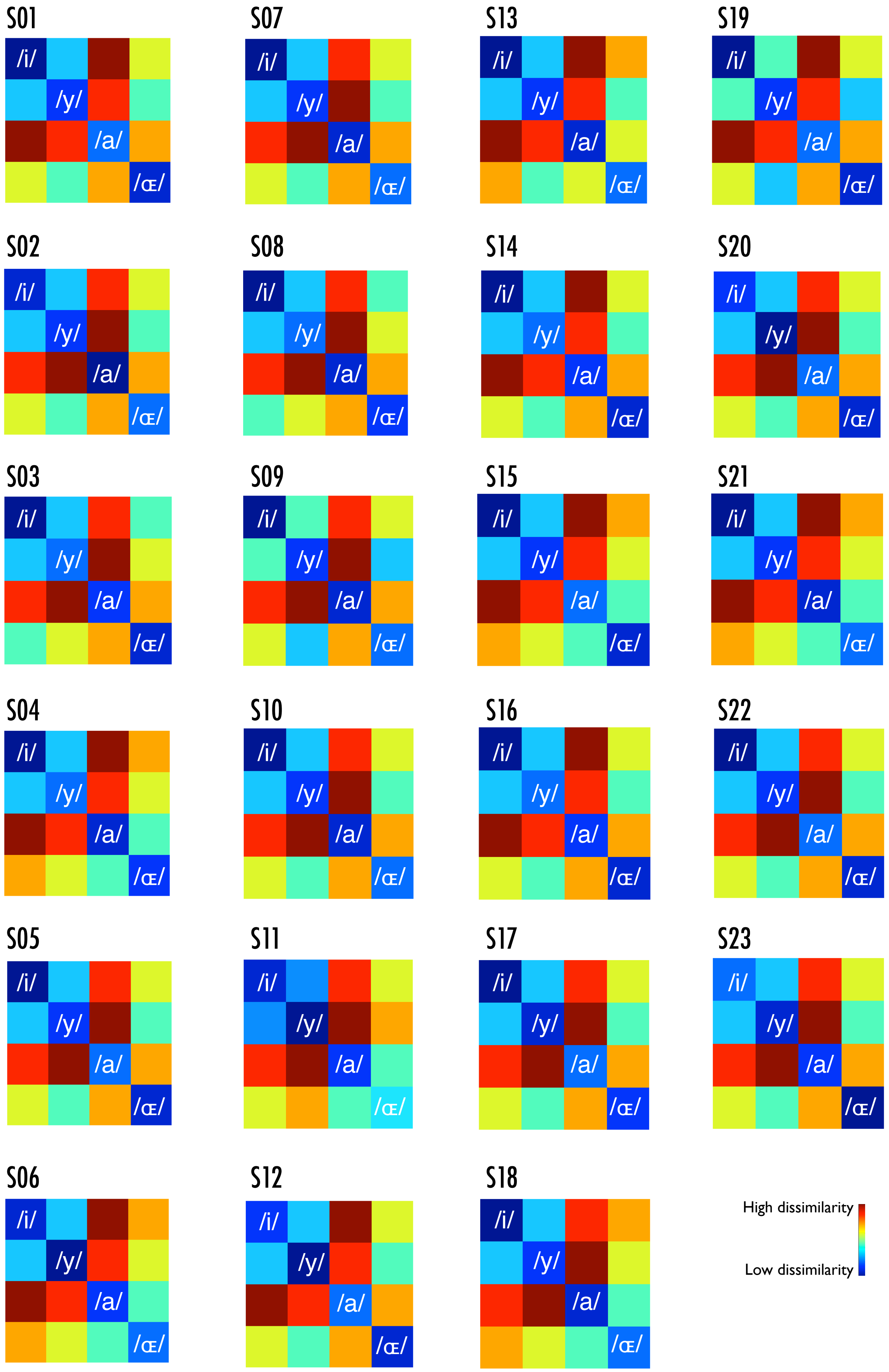

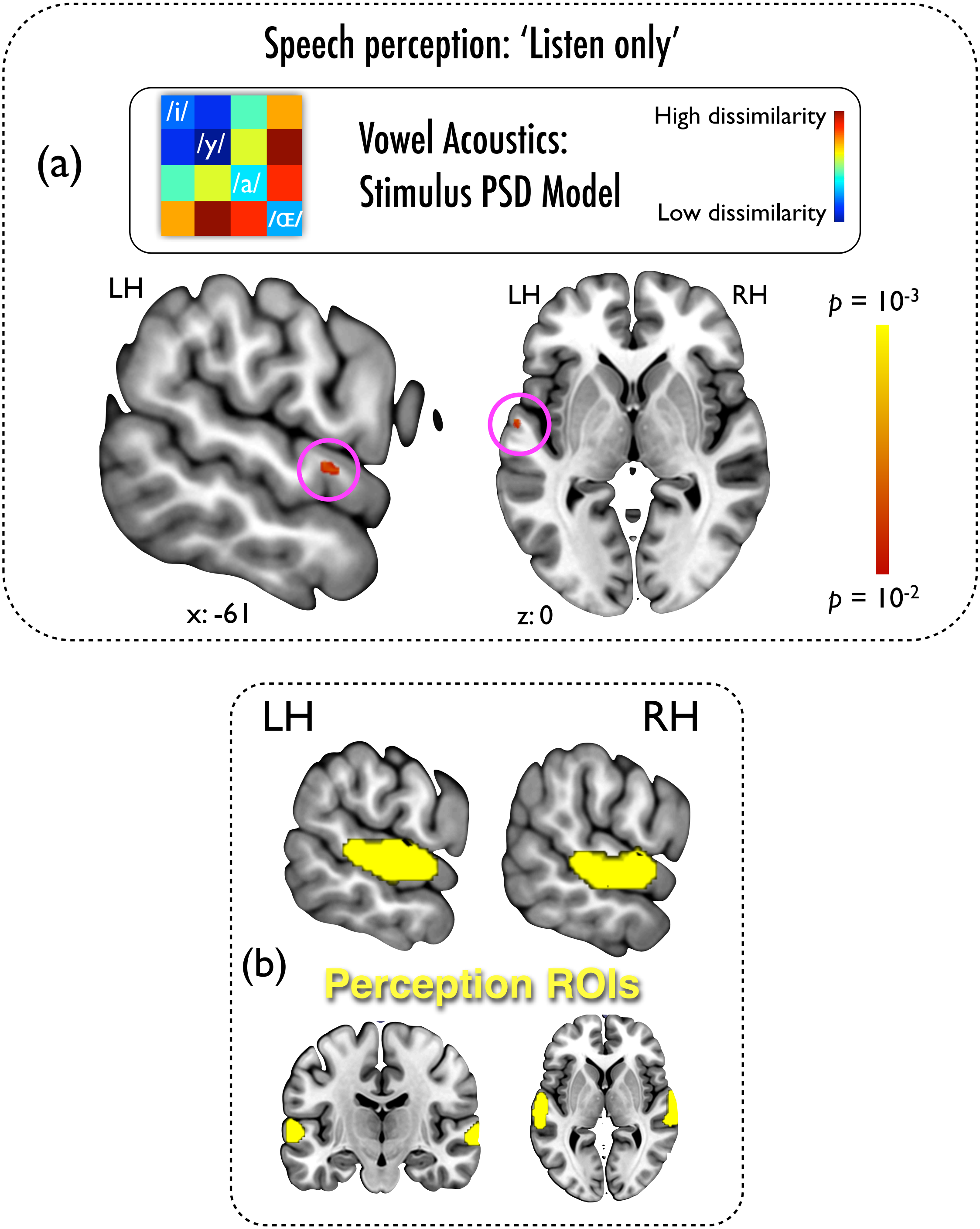
Speech perception: 'Listen only'
(a)
/i/
/y/
/a/
/Œ/
Vowel Acoustics:
Stimulus PSD Model
High dissimilarity
Low dissimilarity
LH
LH
RH
p = 10-3
p = 10-2
x: -61
z: 0
LH
RH
(b)
Perception ROIs
